# Status and Influencing Factors of Physical Exercise among College Students in China: A Systematic Review

**DOI:** 10.3390/ijerph192013465

**Published:** 2022-10-18

**Authors:** Mingzhu Pan, Binbin Ying, Yani Lai, Garry Kuan

**Affiliations:** 1 School of Physical Education, Shangrao Normal University, Shangrao 334001, China; 2 Exercise and Sports Science Programme, School of Health Sciences, Universiti Sains Malaysia, Kubang Kerian 16150, Kelantan, Malaysia; 3 Health Supervision Institute of Shanghai Huangpu District Health Bureau, Shanghai 200011, China; 4 School of Education Science, Shangrao Normal University, Shangrao 334001, China

**Keywords:** physical activity, factors, status, college students, Chinese, systematic review

## Abstract

The status of the physical exercise of college students has been a popular topic in China. This study systematically reviewed the exercise status of Chinese college students and its influencing factors. A keyword and reference search were conducted in the Web of Science, PubMed, Cochrane library, and the China National Knowledge Infrastructure. Additionally, Google Scholar was searched to collect literatures related to physical activity of Chinese university students published in Chinese and English from 1 January 2017 to 30 July 2022. Fifteen studies met the selection criteria and were included in the review. The results show that the main motivation for Chinese college students to exercise is to strengthen their bodies, with running and walking ranking first and ball games ranking second in importance. Most of the college students exercised three times a week, which is the recommended minimum, and most of their workouts were of moderate intensity. Additionally, the workouts lasted for 30 min to 60 min. The main factors affecting college students’ exercise are lack of time due to academic pressure, facilities constraints, and lack of professional exercise guidance. In conclusion, the physical fitness of university students should not be underestimated, and this study provides additional reference to promote healthier lifestyles among Chinese college students.

## 1. Introduction

With rapid social and economic development, people’s awareness of health is increasing. The ‘Healthy China 2030 plan’, which was proposed in 2016, advocates for increasing young people’s passion in sport, ensuring that schoolchildren engage in more than an hour of physical activity daily and the youth become proficient in more than one motor skill [1]. The program, which offers a sound theoretical foundation for student health, specifies that school students should engage in moderate-intensity physical activity more than three times per week and that the national criteria for student physical fitness should be at least 25% excellent. Meanwhile, it has been five years since the Healthy China plan was officially proposed and implemented. Therefore, this study takes this context as an entry point to explore the physical activity status of Chinese university students during this five-year period.

University is the last stage of physical education for students, and how they exercise there has a big impact on whether or not they developed a lifelong interest in sports. University students are generally between the ages of 18 and 23 [2]. Psychologically, they are essentially adults with their own perspectives and thinking. All physical functions have reached their peak in adulthood, and physical abilities like endurance, speed, and explosive power have increased [3]. Meanwhile, it has been proven that exercise can reduce poor mental health, self-harm, and suicidal attempts [4]. In addition, risks for depression, type 2 diabetes, ischemic heart disease, and other illness are linked to physical inactivity [5]. Therefore, the strategies needed to promote physical activity have become an important public health approach for the prevention of chronic diseases. It has been established that adults engaging in regular physical activity will improve their health [6]. Thus, for college students, maintaining good exercise habits can effectively reduce their risk of chronic diseases. Moreover, the academic years are the optimum time for university students to learn the fundamentals of exercise, form the proper participation habits in sports, and cultivate a lifelong sports consciousness.

However, several recent National Youth Physical Fitness Reports showed that the overall physical fitness of college students in China is on a downward trend [7]. The main manifestations are that the students’ amount of exercise decreases year by year, the obesity rate occurs and develops rapidly, the height, body weight, and chest circumference of students increase rapidly, but the lungs capacity and strength quality continue to decline [8]. Meanwhile, college students’ subjectivity is neglected during the process of physical education, and multiple factors cause the lack of awareness of the importance of exercise among university students [9]. Based on this circumstance, it has become an important part of our education to improve and increase university students’ interest towards participating in sports, improve their enthusiasm to participate in sports, increase their awareness to exercise on their own, and enhance their physical fitness.

To understand the current situation of exercise among Chinese university students and its influencing factors, and to provide a reference for promoting the development of a healthy lifestyle among university students, this study conducted a systematic review from relevant Chinese and English literature on exercise among Chinese university students published in five years from 1 January 2017 to 31 July 2022. We aimed to identify (a) the exercise status of college students in China and (b) influencing factors of exercise among college students in China.

## 2. Materials and Methods

### 2.1. Eligibility Criteria

Inclusion criteria: (1) Research design: cross-sectional studies, interventions or experiments, retrospective or prospective cohort studies; (2) Subjects: College students; (3) Outcomes: the research investigated the statistical relationships with variables and provided a measurement of physical activity as the dependent outcome. (4) Country/area: mainland China; (5) Type of article: peer-reviewed articles; (6) Time range: published from 1 January 2017 to 31 July 2022.

Exclusion criteria: (1) Article types are pure overviews, news, press releases, reviews, and conference bulletins; (2) Unavailable full text; (3) Insufficient data; (4) Published before 2017.

### 2.2. Search Strategy

A keyword search was performed in the four electronic bibliographic databases: Cochrane Library, PubMed, Web of Science, and the CNKI (China National Knowledge Infrastructure). Additionally, we also conducted hand-searching in Google Scholar. The search algorithm included all possible combinations of keywords from the following two groups: (1) “exercise”, “exercises”, “physical activity”, “physical activities”, “ motor activity”, “ motor activities”, “fitness”, “workout”, “physical fitness”, “active lifestyle”, “active lifestyles”, “sports”, “sport”; (2) “Chinese college students”, “Chinese college student”, “Chinese university students”, “Chinese university student”, “Chinese undergraduates”. The search algorithm in Pubmed is provided upon request. After identifying the included articles, a reference search and a citation search were conducted on the included papers, which were then screened and evaluated using the same literature screening criteria to determine if there was any new included literature, and the reference search and citation search were repeatedly screened and evaluated for all newly included articles until no new relevant paper was included.

### 2.3. Study Quality Assessment

This study used the National Institute of Health’s Quality Assessment Tool for Observational Cohort and Cross-Sectional Studies to assess the quality of each included study. The evaluation tool has 14 criteria. For each criterion, a score of one was assigned if “yes” was the response, whereas a score of zero was assigned otherwise (i.e., an answer of “no”, “not applicable”, “not reported”, or “cannot determine”). The total scores range from 0~14.

Standardized data extraction was used to collect the following methodological and outcome variables from each included study: author(s), year of publication, research area, study design, sample size, exercise motivation, exercise frequency, exercise duration, exercise program, exercise site, exercise form, and the related influencing factors, etc. The data extraction was independently conducted by two reviewers (M.P. and Lai) in this review. When there is disagreement, a third researcher (Ying) decides further and identifies the paper that needs to be read in full, and then the two researchers jointly determine the final literatures to be included in the study through discussion.

## 3. Results

### 3.1. Basic Characteristics of the Included Papers

**Figure 1:**
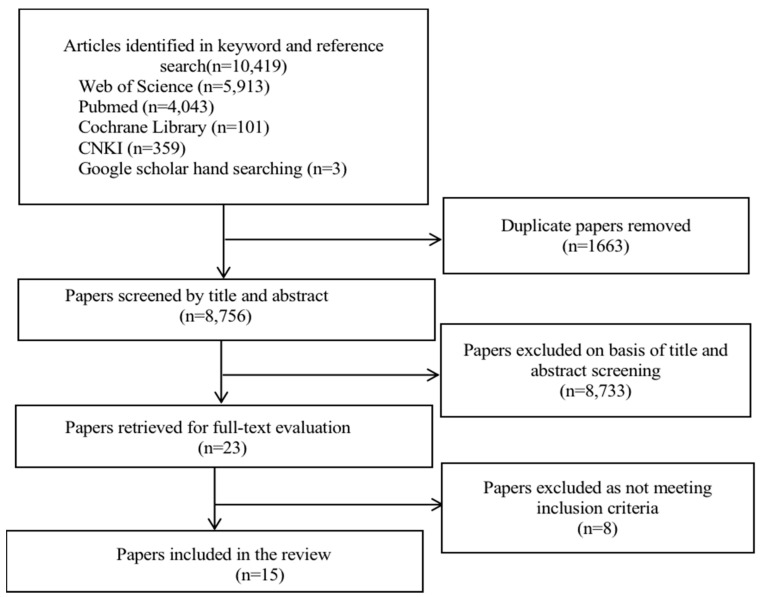
Study selection flowchart.

Figure 1 shows the study selection flowchart. We identified a total of 10,419 articles through keyword and reference searches, including 10,060 papers in English and 359 papers in Chinese. Overall, there were 5913 articles from Web of Science, 4043 articles from PubMed, 101 articles from Cochrane Library, 359 articles from CNKI, and three papers from Google Scholar through hand-searching. Based on the inclusion and exclusion criteria, 15 articles (six papers in English and nine papers in Chinese) were finally included (Figure 1).

Of these, three articles were studied at Beijing universities [10,11,12], two at Shanghai universities [12,13], two at Guangdong universities [14,15], one at Shandong universities [16], one at Anhui universities [17], one at Hubei universities [18], one at Henan universities [19], one at Guangxi universities [20], and one at Guizhou universities [21], while the rest did not clearly show the name of the university or the area in which the university is located [22,23,24]. Two eastern universities, two southern universities, four northern universities, and four central universities remained unspecified. All of the selected papers were published after 2017, with two published in 2017, three in 2018, one each in 2019 and 2020, six in 2021, and two in 2022. The research design of all the 15 articles was cross-sectional, with sample sizes ranging from 200 to 1512, nine articles in the 100–999 range, and six articles in the 1000–9999 range, and with an average of 864, a median of 929, and a standard deviation of 447.8. In addition, the majority of studies were based on subjective self-report surveys. The proportion of females in the sample ranged from 26.7% to 50.5%.

The quality of the literature score ranged from a minimum of 7 to a maximum of 12, and the mean score of the included articles’ quality assessment was 9.26. All the 15 papers included research questions and objectives that clearly specified and defined the study population, with a participation rate of more than 50%. The sample size attrition rate for 14 articles was less than 20%, while it was not indicated in one paper. Participants were recruited from the same or similar populations during the same period, with uniform inclusion and exclusion criteria pre-defined for all potential participants (See Table 1).

### 3.2. Exercise Status of College Students in China

Exercise motivation: We found that the primary factor in university students’ motivation to exercise was to improve their physical fitness, which is in line with the results of the National Sports Administration’s National Fitness Survey [25]. Among the 15 articles, 14 showed that physical exercise strengthens the body, followed by relieving academic pressure.

Exercise frequency: The General Administration of Sports of China, in its analysis of the “Survey Communique on the Status of National Fitness Activities in 2020”, concluded that the average physical activity population in China is 37.2 %. In this study, physical activity was measured as exercise ≧ three times/week, and the percentage of physical activity among university students was found to be ≧ 37.2% in 10 out of 15 papers, which reached the national average [25].

Exercise duration: Among the 15 articles, eight papers showed that university students’ physical exercise lasted for ≧30 min per exercise session, which met the standard criteria for physical exercise length [25].

Exercise program: University students’ most popular exercise activities were mainly running and walking, followed by ball games. There were differences by gender and by grade. Li et al. [15] found that 74.58% of university students mainly participate in aerobic exercise and endurance sports, with a higher proportion of runners, accounting for 79.16%. This indicates that running is still a popular sport among university students. Ai [20], Cao and Zhao [10], and Qin [17] revealed that running was the most popular sport among university students, followed by basketball.

Exercise site: The most popular exercise areas for university students were the public sports grounds, athletic fields, and basketball courts. Eleven of the fifteen papers indicated that the most popular sites for exercise were athletic fields, basketball courts, and finally gymnasiums. Hence, it can be concluded that the most popular places for exercise are the public sports grounds on campus.

Group or individual: According to the study’s findings, nine out of fifteen papers indicated that college students would choose to exercise alone. Qin [17] showed that 56.6% of the respondents prefer to walk on their own. Additionally, the findings of Ai [20] also clearly indicate that 42.6% of university students participate in extracurricular physical activity on their own (See Table 2).

### 3.3. Influencing Factors of Exercise among College Students in China

Academic pressure: This study found that some university students reported high academic pressure and lack of time as their reasons for giving up physical exercise, with nine of the papers mentioning this factor. Five articles mentioned the lack of time to exercise due to the pressure of studying, senior examinations, and employment internships. Although university students know that exercise is good for their health, they must give it up because of time constraints. In recent years, due to the COVID-19 pandemic and the economic downturn, university students have been under great pressure to find employment, which has led to a rise in taking examinations for higher studies [26].

Restrictions on venues and facilities: Exercise venues, surroundings, and facilities were the secondary factors influencing college students’ workouts. The preferred place for college students to exercise is the free playground or sports center on campus, but some studies showed that they are inadequate. Wang et al. [27] stated that insufficient venues are the main barrier to university students’ physical exercise, followed by a lack of time and organization. Cheng and Zhu [18] claimed that due to the college expansion plan and the increasing number of students in universities, the supply of sports venues and sports facilities is insufficient, which restricts the students from exercising when they want to.

Lack of professional guidance: This study found that the availability of professional exercise instruction was also an important factor in determining the university students’ exercise participation. Of these, seven articles reported that the lack of professional exercise guidance was one of the factors that led the college students not to participate in physical exercise. For instance, some university students have specific exercise goals to achieve (such as weight loss, muscle building, etc.), but due to the lack of professional exercise guidance, they assume that self-exercise will not help them reach their objectives. As a result, they either stop exercising altogether or wait for a chance to be guided to exercise.

## 4. Discussion

For this review, we identified nine papers published in English and six papers published in Chinese and summarized the status and factors influencing physical activity among Chinese college students. This paper reviews the research design of the articles in this area and the characteristics of physical activity among university students in terms of exercise motivation, exercise frequency, intensity, exercise programs and gender differences, exercise venues, etc. According to the findings of this systematic review, the main factor among university students’ exercise motivation was to strengthen their physical health. The majority of the college students in China exercised more frequently than 3 times/week, with each workout regularly lasting between 30 and 60 min, which reached the national average [25]. Given that effective and regular exercise can effectively prevent chronic diseases, it has been suggested that for 20~64 year old, the rate of chronic pain was 10~12% lower for those exercising 1~3 times a week for at least 30 min duration or of moderate intensity, relative to those not exercising [28]. Besides, university students prefer moderately intense sports as well as aerobic exercises like jogging and ball games and they frequently go to the free sporting facilities on campus, like the basketball court and athletic field [29,30]. The main type of exercise for university students is mainly alone, which is in line with the psychological characteristics of college students to pursue themselves and their individuality [31].

The main factors affecting university students’ exercise are lack of time due to academic pressure, facility constraints, and lack of professional exercise guidance. Academic pressure was identified as one of the main factors for the lack of time for exercise, which reflects the concern among students that exercise may hamper academic performance. However, a previous study revealed that those who frequently engaged in exercise had a significantly higher GPA [32]. This suggests that education is needed to enhance the awareness of the benefits of exercise in university life. Meanwhile, Liu et al. [33] indicated that for college students, they should have positive thoughts and good behavior when engage regularly in physical activity, which inspires them to engage in physical activity with passion. For facilities constraints, a study in Greek indicated that PE teachers who work in schools with very satisfactory sport facilities seem to be more satisfied in comparison to PE teachers who work in poor sport facilities [34]. In the same way, due to sport facilities and site constraints, students may be less enthusiastic about sports. Therefore, whether the exercise facilities are fully equipped, and the sports centers are sufficient is the embodiment of measuring the physical exercise culture in colleges and universities. Only by adequately providing the necessary sports facilities can more college students be attracted to participate in physical activity, better forming the sports culture. Another key element influencing university students’ exercise or fitness is a lack of professional exercise guidance. In China, the training and accreditation of rehabilitation therapists and exercise prescription practitioners are under development [35]. Currently, there are no sport instructors on campus [36]. As a result, college students may give up exercise or working out since they do not know how to perform an efficient and scientific method of exercise.

Finally, there are several new findings in this systematic review, yet it has certain limitations. One limitation in this review is the research design, as all of the included papers were cross-sectional studies and it is not suitable to make causal inferences. Another limitation is the research content, as no more than two papers were found in the included literature for quantitative analysis of their corresponding variables, so we could not conduct a meta-analysis.

This is the first study to examine the physical activity levels of Chinese university students systematically. In addition, five years have passed since the official commencement of the Health China Plan. Therefore, numerous international researchers are interested in the transition preceding and during the implementation of the Health China Plan. However, another limitation of this study is that it was designed specifically for the Chinese population. Due to the vastness of China’s geographical area, the study is quite unique and robust. Due to cultural and political differences, the results should be read with caution, as they may not be applicable to other nations.

## 5. Conclusions

According to the results of our study, college students’ participation in sports and physical activity seems to be relatively good. Most indicators, such as exercise frequency, exercise intensity, exercise duration, etc., are in compliance with national standards. However, there are still some factors that restrict university students from participating in sports. Based on that, at the government level, we recommend that more funds be invested in establishing university stadiums and improving sports facilities to guarantee the basic conditions for college students’ exercise. On the other side, at the school level, in order to actively encourage students to exercise, schools may adopt measures like relating daily exercise to final grades. In addition, the school may recruit some sports instructors in sports centers or gyms to provide professional guidance to students who have questions about exercise.

## Figures and Tables

**Table 1:** Basic information of included studies.

Authors	Research Area/School Name	Study Design	Sample Size	Female (%)	Sample Turn-Over Rate	Study Quality Assessment Score
Cao and Zhao (2017) [10]	USTB (University of Science and Technology Beijing)	Cross-sectional study	200	N/A	6%	10
Sun, (2017) [14]	Guang Dong province/South China University of technology (SCUT)	Cross-sectional study	313	N/A	24.61%	11
Cheng and Zhu (2018) [18]	15 universities in Hubei province	Cross-sectional study	1000	40.5%	4.1%	11
Wang, (2019) [11]	Beijing/Tsinghua University	Cross-sectional study	1500	42.1%	5.8%	10
Wang and Zhai, (2018) [16]	Shan Dong province/Lin yi university	Cross-sectional study	282	N/A	4.7%	7
Chen, (2019) [19]	Henan University of Science and Technology, Luoyang Normal University, Luoyang institute of science and technology	Cross-sectional study	1151	49.1%	1.5%	10
Dai et al. (2020) [21]	10 universities in Guizhou province	Cross-sectional study	1276	n/a	2.1%	9
Ai, (2021) [20]	Guangxi province	Cross-sectional study	900	N/A	2.7%	8
Jiang et al. (2021) [13]	Shanghai Jiao Tong University	Cross-sectional survey	1512	26.7%	1.4%	8
Qin, (2021) [17]	An Hui province	Cross-sectional study	929	50.5%	7.1%	12
Li et al. (2021) [15]	10 universities in Guangzhou province	Cross-sectional study	800	48.5%	2.12%	9
Lyu et al. (2022) [23]	N/A	Cross-sectional study	396	N/A	1%	7
Peng and Tang, (2021) [22]	N/A	Cross-sectional study	321	N/A	3.74%	10
Zhang et al., (2021) [12]	Beijing and Shanghai	Cross-sectional survey	1406	47.8%	N/A	8
Zhang et al. (2022) [24]	N/A	Cross-sectional study	976	46%	2.4%	9

N/A = no answer.

**Table 2:** Contents of the studied in review.

Study ID	Exercise Motivation	Exercise Frequency, Exercise Duration		-Exercise Program -Intensity	Difference (Male/Female)	Exercise Site	Group or Individual
Cao and Zhao (2017) [10]	Improve physical fitness	Male: 34%, ≧3 times/week; Female: 27.6%, =1 time/week. Male: 43.2%, 30~60 min. Female: 71.8%, ≦30 min		-Male: running, basketball Female: Walking, badminton. -N/A	Y	Track and field	N/A
Sun, (2017) [14]	Keep fit	22.1%, ≧3 times/week; 13.1%, =3 times/week; 31.7%, =2 times/week.		-Male: basketball, badminton, run, swimming Female: badminton, run -N/A	Y	Sports hall, athletic field	Depend on program
Cheng and Zhu (2018) [18]	Male: 34.62%, keep fit; 33.61%, personal habit; Female: 33.83%, keep fit, 32.1%, personal habit	Male: 41.85%, =1~2 times/week. 26.72%, 3~4 times/week. Female:52.84%, 1~2 times/week. 20.49%, 3~4 times/week Male:40.84%, 30 min~60 min. 32.27%, 60 min~90 min. Female: 42.72%, 30 min~60 min; 30.37%, 60 min~90 min.		-47.5%, ping-pang. 42.8%, badminton. 44.1%, tennis; -N/A	Y	Free sports center at campus	31.70%, individual. 28.8%, group
Wang and Zhai, (2018) [16]	Keep fit	N/A	63%, ≧30 min; 11%, ≦30 min;	-36.2%, Running; 23.5%, basketball. -N/A	N/A	45.1%, Free sports center at campus	individual
Chen, (2019) [19]	Keep fit	37.3%, ≧3 times/week.		-N/A -Males: vigorous intensity exercise. Female: moderate intensity	Y	Free sports center at campus	N/A
Wang, (2019) [11]	Physical fitness	Male, 75.9% ≦ 3 times/week. Female, 75.9 %≦ 3 times/week.		-N/A -Males: vigorous intensity exercise. Female: moderate intensity	Y	Athletic field	individual
Dai et al. (2020) [21]	Improve physical fitness	39.9%, 1~2 times/week. 29.9%, 3~4 times/week. 27%, ≧5 times/week. Mainly range from 31~60 min (41.3%)		-Running, basketball, walking, badminton. -50.3%, male = Moderate intensity exercise; 42.3%, female = Moderate intensity exercise	Y	87.7%, Free sports center at campus	55.09%, individual
Ai, (2021) [20]	Improve physical fitness	49.7%, =3–4 times/week; 16.3%, = 5 times/week. Mainly range from 30–60 min.		-Male: basketball, running; Female: running, fitness course -N/A	Y	Track and field, basketball court	individual
Jiang et al. (2021) [13]	Improve mental health	N/A		-N/A Vigorous Physical activity	Y	N/A	N/A
Qin, (2021) [17]	Relieve academic pressure and keep fit	52.6%, Proportion of regular participants in physical exercise Male: 54%, no fixed duration; female: 42.3%, no fixed duration		Male: basketball, running, walking Female: walking, running, ping-pang -N/A	Y	Track and field, basketball court	56%, individual
Li et al. (2021) [15]	78.29%, improve physical fitness	50%, =2~3 times/week; 31.05%, =4~5 times/week; 10.86%, ≧6 times/week; Mainly range from 30~60 min		74.58%, aerobic exercise and endurance exercise; 56.58%, antagonism sports; 39.34%, dance items. -N/A	Y	Free sports center at campus	individual
Peng and Tang, (2021) [22]	Keep fit, =78.29%; Actively participate in physical exercise, =51.98%	49.55%, =2~3 times/week. 31.03%, =4~5 times/week; 10.86%, ≧6 times/week. Mainly range from 30~60 min.		Male: dangerous coping behaviors, female: resist coping behaviors Moderate-intensity 50% N/A	Y	N/A	individual
Lyu et al. (2022) [23]	Improve physical health and improve the results of physical test	45.2%, occasionally perform physical exercise; 19.9%, regular perform physical exercise		N/A	N/A	Free sports center at campus	N/A
Zhang. et al. (2022) [24]	Improve mental health quality	18.9%, =2 times/week. 32.8%, =3 times/week. 33.6%, =31~45 min. 16.5%, =46~60 min.		-Medium exercise, 46.54%: Boys, ME = 53.71%, EE = 35.62%; Girls, ME = 37.47%, SM = 50.78%	Y	N/A	individual
Zhang et al., (2021) [12]	Perceive health	N/A		-N/A Moderate to vigorous physical activity	N/A	N/A	N/A

Y = yes; N/A = no answer.

## Data Availability

The data used are available within the manuscript.
